# The Bedrock technique with triangular titanium rods for spinopelvic fixation and sacroiliac joint fusion: is there a learning curve?

**DOI:** 10.1007/s00590-025-04431-0

**Published:** 2025-07-23

**Authors:** Cale J. Hendricks, Jason J. Haselhuhn, Paul Brian O. Soriano, James T. Longhurst, Christopher T. Martin, Jonathan N. Sembrano, Nathan R. Hendrickson, Kristen E. Jones, David W. Polly

**Affiliations:** 1 Department of Orthopedic Surgery, University of Minnesota, Minneapolis, MN USA; 2 Department of Neurosurgery, University of Minnesota, Minneapolis, MN USA

**Keywords:** Spinopelvic fixation, Lumbosacral fusion, Sacroiliac joint, Triangular titanium rod, Porous fusion/fixation screw, Spinal deformity

## Abstract

**Purpose:**

The Bedrock technique involves fusion of the sacroiliac (SI) joint at the time of long construct spinal fusion extending to the pelvis. This is done with triangular titanium rods (TTR) placed parallel and cephalad to S2AI screws, requiring placement of two implants into the small bony corridor in the teardrop of the pelvis. In our initial 21 cases adopting this technique, we found a 7.1% rate of implant malposition requiring intraoperative repositioning. The objective of this follow-up study was to report on our implant malposition rates over a larger number of cases, to determine a potential learning curve, and to report on strategies for minimizing implant malpositioning.

**Methods:**

Surgeries in which SI joint fusion was performed concomitant with spinopelvic fixation using the Bedrock Technique with TTRs and CT-navigation between 5/1/2019 and 5/27/2021 were reviewed. Operative reports were analyzed and any intraoperative TTR/S2AI screw malpositions were recorded. Demographic/surgical information, TTR/S2AI specifications, and PROMs were obtained and analyzed along with risk assessment and correlation of failure between cohorts (α < 0.05).

**Results:**

A total of 43 patients were included with a mean length of fusion of 4.8 (1–14 levels). Overall, 86 TTRs and S2AI screws were implanted of which, 4/86 (4.7%) TTRs were repositioned intraoperatively and 1/86 (1.2%) was removed and replaced with allograft, while 0/86 (0%) S2AI screws were malpositioned. All TTR malpositions were medial and/or cephalad. In the initial 21 cases, 3/42 (7.1%) TTRs required intraoperative repositioning; this decreased in the subsequent 22 cases to 2/44 (4.5%) with a non-significant relative risk of 1.57 (*p* = .664). Malposition was non-significantly negatively correlated to time (r = -.135; *p* = .39). Our patients showed statistically significant (*p* < .001) one-year improvements in mean ODI, VAS back, and PROMIS-10 metrics, meeting MCID for ODI and VAS back.

**Conclusions:**

Posterior placement of a TTR above an S2AI screw poses technical challenges due to the natural cephalad narrowing of the bony corridor in the teardrop and blunt tip of the implant which predisposes it to medial skiving as it is passes through the SI joint. Our overall intraoperative reposition rate for caudal S2AI screws was 0% compared to 5.8% for cephalad TTRs, which decreased from 7.1 to 4.5% between our first- and second-year cohorts, suggesting a potential learning curve. All malpositions occurred medially and/or cephalad, and over time we transitioned our technique to placing implants as low as possible in the teardrop to minimize this risk.

## Introduction

Spinal fusion extending to the sacrum increases stress on the sacroiliac (SI) joint [1], and studies have shown an increased rate of new-onset SI joint symptoms with increased length of fusion [2]. A prospective randomized multi-centered study reported a 16% prevalence of preoperative SI joint pain, while the rate of new-onset SI joint pain after lumbosacral fusion was reported to vary from 3 to 90% (mean 24%) by a systematic review [3, 4]. Furthermore, pelvic fixation was found to increase the incidence of SI joint pain by 5.9 times compared to constructs without pelvic fixation [3]. The SI joint contains substantial proprioceptive and nociceptive nerve fibers [5] and is mobile with an average rotation range of motion of ± 2° with some literature reporting up to 9° [6]. Long construct lumbosacral fusion has been shown to increase biomechanical stress and force moments to the SI joint and pelvic anchoring construct [1]. This, in turn, may contribute to joint dysfunction and pain with motion [7]. In response to these concerns, some surgeons have begun performing SI Joint fusion at the time of spinal fusion cases that extend to the pelvis.

The Bedrock technique involves concomitant SI joint fusion and spinopelvic fixation performed with porous triangular titanium rods (TTR) placed cephalad to sacral-2-alar-iliac (S2AI) screws from a starting point on the dorsal sacrum and extending into the teardrop portion of the bony ilium [8, 9]. A finite element analysis verified that SI joint fusion devices significantly decrease SI joint range of motion and that TTRs implanted posteriorly can elicit bony fusion of the joint [10, 11]. Additionally, further analysis and mechanical tests of this construct demonstrated lower S1 pedicle and S2AI pelvic screw bending movements in all directions, which provided a protective effect on S1 and S2AI screws compared to pelvic fixation with S2AI screws alone [10, 12]. This, in return, could potentially decrease the risk of new-onset SI joint pain and pelvic fixation failure, which has been reported to be 22.1% on average (3–41%) [13]. Higher quality data specific to the Bedrock Technique are pending from a prospective randomized multi-center study (SILVIA, NCT04062630; clinicaltrials.gov). Despite the positive biomechanical effects of this device, the surgical technique for proper positioning can be challenging. In our initial 21 cases using this technique, a moderately high rate of implant malposition (7.1%) requiring intraoperative repositioning was reported [9].

Given the novelty of this fusion device, no studies to our knowledge have investigated if the Bedrock technique, specifically TTR implantation and positioning, is associated with a learning curve. Therefore, the objective of this study was to comment on our experience and strategies for performing open fusion of the SI joint with concomitant spinopelvic fixation using TTRs as our experience has grown. In addition, we investigated a possible learning curve at our institution by comparing the rates of intraoperative TTR repositioning between our initial (first-year) and subsequent (second-year) patient cohorts. We predicted a slight decline in malposition rates over time, potentially indicating a learning curve.

## Methods

**Fig. 1:**
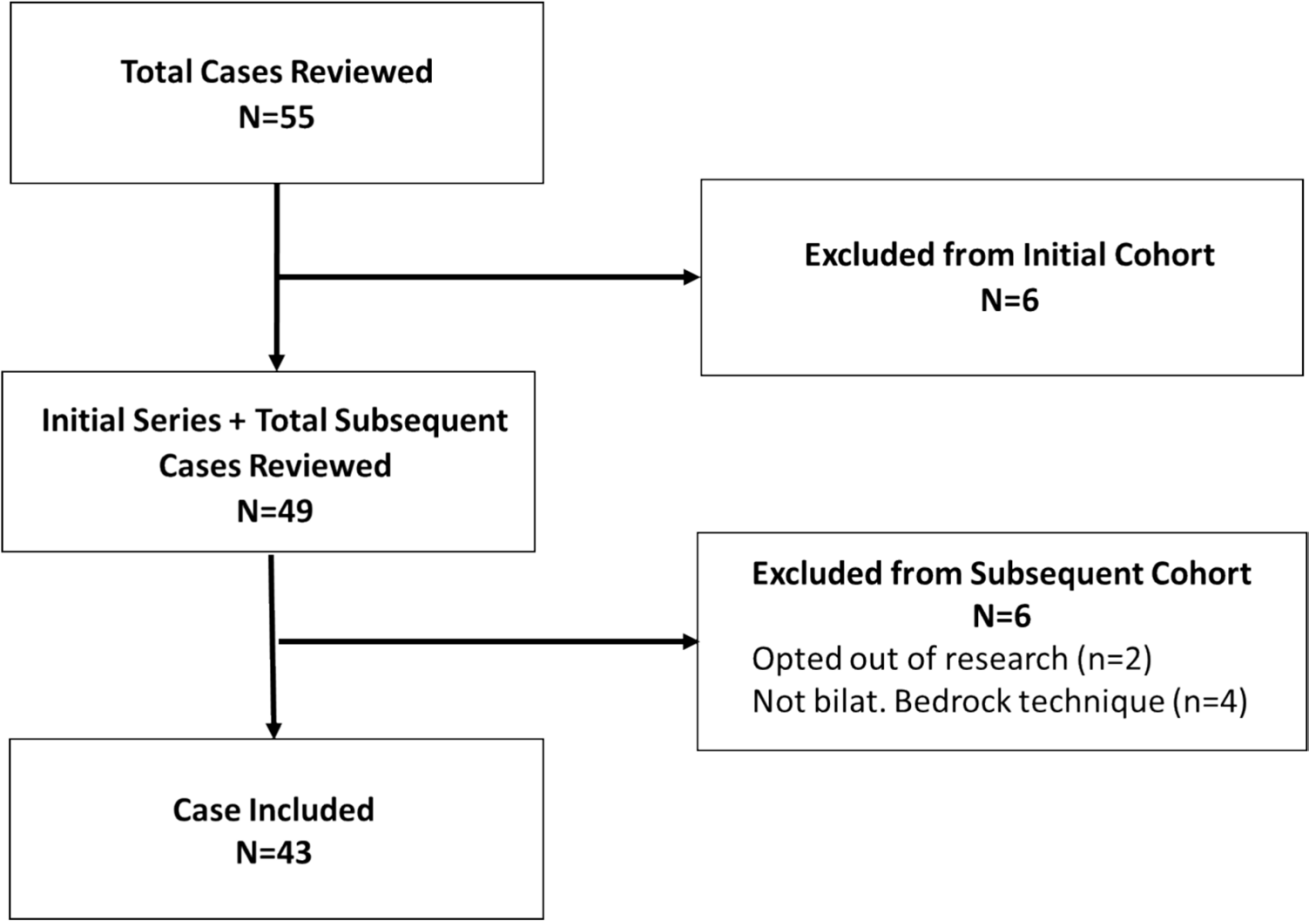
Methodology inclusion/exclusion flow diagram

This retrospective study was approved by the Institutional Review Board prior to data collection. A list of surgeries performed by five participating surgeons between 5/1/2019 and 5/27/2021 were collected, and patients were screened according to the inclusion and exclusion criteria (Fig. 1). Operative reports were reviewed and any intra- or post-operative TTR or S2AI repositions were recorded.

**Fig. 2:**
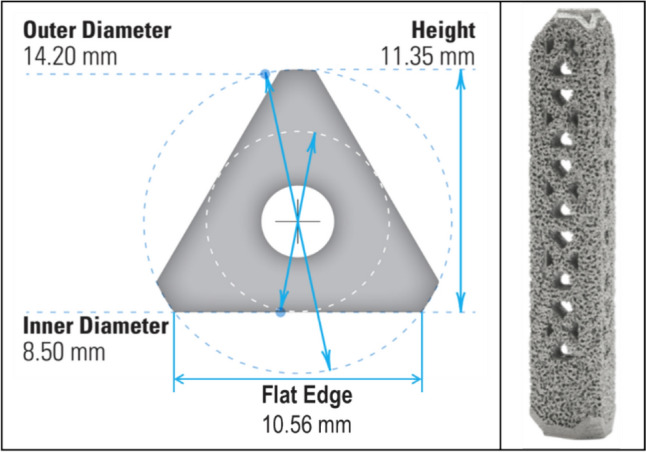
Descriptive Image of the 7 mm triangular titanium rods used in the Bedrock technique

Patients were included if they were ≥ 18 years old and received attempted bilateral SI joint arthrodesis with concomitant lumbosacral fusion surgery using the Bedrock technique with triangular titanium rods (TTR) (iFuse Bedrock, SI-Bone, Santa Clara, CA). The TTR is defined to have a 7mm diameter per the manufacturer (Fig. 2). However, the true inner inscribed diameter of the TTR is 8.5mm given the addition of a 0.75 mm thick titanium plasma spray (TPS) porous layer on the surface. Patients were excluded if they did not consent to the use of the medical records for research, did not receive attempted bilateral surgery using the Bedrock technique, or were not included in the initial series [9].

Demographics (gender, age, BMI, height), surgical information, and implant specifications were obtained. Patient reported outcomes measures (PROMs), including Oswestry Disability Index (ODI), visual analog scale (VAS) for back pain, and patient-reported outcomes measurement information system (PROMIS-10), were collected when available preoperatively and 1-year post-operatively. Minimal clinical important difference (MCID) was defined to be ≥ 13 for ODI, ≥ 2 for VAS, and ≥ 4 for PROMIS.

### Surgical technique

**Fig. 3:**
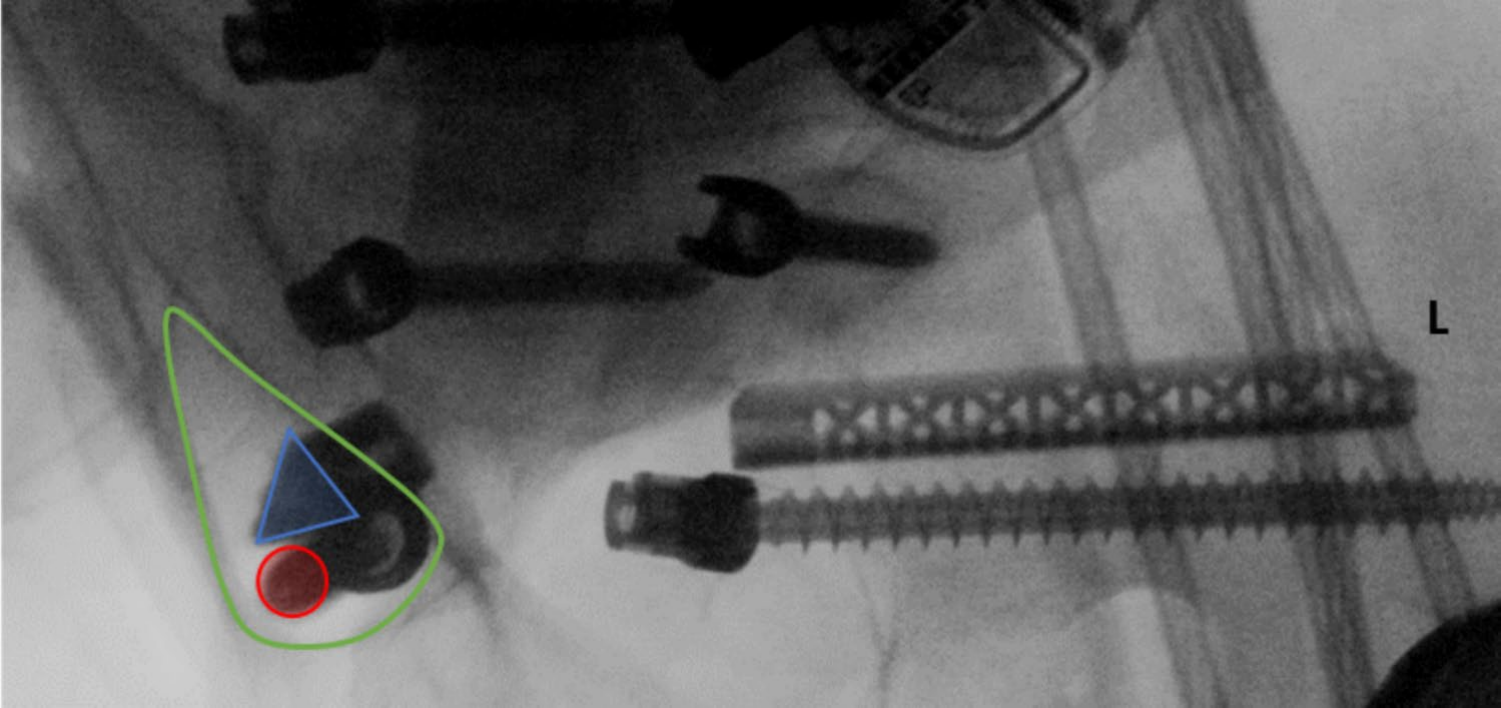
Fluoroscopic AP teardrop view (outlined in green) of an ideally placed left caudal S2AI screw (red circle) and left cephalad TTR (blue triangle)

The surgical technique utilized in this cohort of patients was further described with additional descriptive intraoperative imaging in previous studies [8, 9]. A midline incision was made with bilateral subperiosteal exposure following standard protocol. Subsequent placement of bilateral pedicle and caudal S2AI pelvic screws was performed. Intraoperative CT-based (O-Arm) computer navigation (Medtronic, Minneapolis, MN) was used for all implants. The S2AI screws were started in the distal one-third of the bony bridge between, and just lateral, to the S1 and S2 foramina with a trajectory into the caudal most aspect of the targeted radiographic teardrop (Fig. 3).

**Fig. 4:**
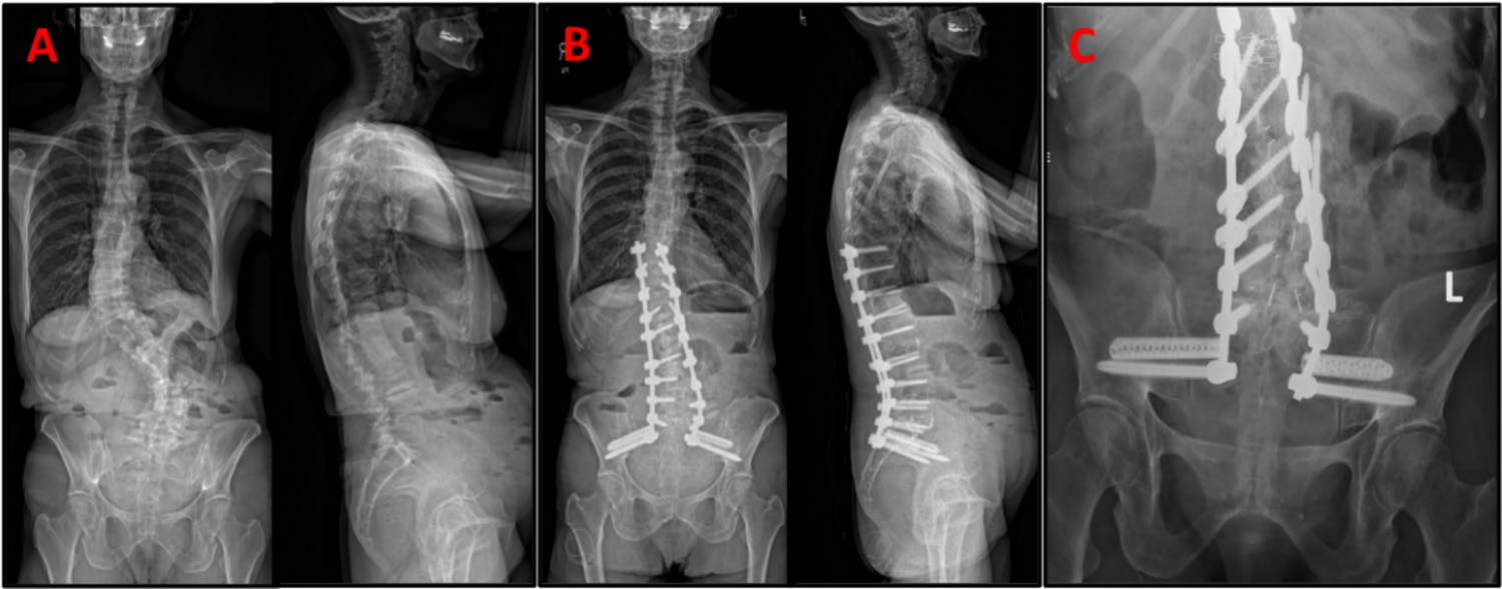
Pre- and post-operative radiographs of a successful deformity correction using the Bedrock technique: **a** full body biplanar (posteroanterior (PA) & lateral) EOS scan three-months pre-operative, **b** repeat EOS imaging one-month post-operation of a T10 to pelvis spinal fusion, **c** Ferguson view offers a true Anteroposterior (AP) of the pelvic for better implant visualization post-operation

Bilateral 3D-printed porous TTRs were placed just cephalad (2–3 mm) and parallel to the S2AI screws allowing for full containment in the teardrop of the ilium. The starting point was marked with a navigated awl, and a pilot hole was drilled with navigated. A reverse threaded guide pin was inserted into the tract and drilled into the distal lateral iliac cortex to prevent backing out. A cannulated drill bit was then used to drill over the guide pin, breaching the SI joint. Further broaching with navigation created a tract with the flat side of the triangular-shaped broach aimed toward the caudal S2AI screw to minimize separation. The greatest length TTRs were selected, passed over the guide pin, and impacted until the appropriate depth was reached. Additional confirmatory intraoperative CT scans and fluoroscopic radiographs were obtained to verify implant position, and in the presence of a cortical breach, the implant was removed and appropriately repositioned. Once the implant position had been verified, the remainder of the surgery proceeded following standard techniques. Overall instrumentation was assessed with pre-and post-operative imaging (Fig. 4a, b), including a Ferguson view of the pelvis (Fig. 4c).

### Statistical analysis

Descriptive statistics and frequencies were calculated for demographics, surgical procedures, and PROMs. Statistical analysis was performed using SPSS version 29.0 (IBM Corporation, Armonk, NY). Independent sample t-tests were used to determine differences in demographics, surgical procedures, and changes in PROMs (pre/post) between the first-/second-year cohorts and between yes/no malposition cases. Paired sample *t*-tests were used to assess PROMs between pre- and post-operative time points. Pearson correlation tests were used to look at malposition over time since the Bedrock technique was introduced at our institution, and chi-squared tests were performed for risk assessment between cohorts. The threshold for significant difference was *α* < 0.05.

## Results

The final cohort included 43 patients (14 male, 29 female) with a mean age of 62.3 ± 11.0. Average body mass index (BMI) and height were 31.5 ± 7.1 kg/m^2^ and 1.7 ± 0.1 m, respectively. Upper instrumented vertebrae (UIV) ranged from L5 to T4 with a range of 1–14 levels fused and a mean of 4.8 (Table 1). Surgeries were performed by a co-surgeon team in 28 cases and a single surgeon in 15 cases. We found no difference in patient demographics or surgical details between the initial and subsequent cohorts (Table 1) or between patients with and without repositioned TTRs.

**Table 1:** Combined, initial, and subsequent patient cohorts’ demographics and surgical data

	Combined (mean ± SD)	Initial (mean ± SD)	Subsequent	*p* value
No. patients	43	21	22	–
Sex (M:F)	14:29	6:15	8:14	–
Age (years)	62.3 ± 11.0	61.5 ± 11.1	63.0 ± 11.1	.665
BMI (kg/m^2^)	31.5 ± 7.1	30.9 ± 7.5	32.0 ± 6.8	.602
Height (m)	1.7 ± 0.1	1.7 ± 0.1	1.7 ± 0.1	.780
No. levels fused	4.8 ± 2.8	4.9 ± 2.0	4.6 ± 3.3	.753
No. levels instrumented	7.0 ± 2.8	6.9 ± 2.0	7.0 ± 3.4	.872
UIV	T4-L5	T10-L4	T4-L5	–
No. TTR	86	42	44	–
TTR Malposition	5	3	2	0.664

^**^p value < 0.01; *p value < 0.05

Overall, 86 TTRs were implanted with a single diameter and lengths of 65–90 mm (Table 2). Of these, 5/86 (5.8%) were malpositioned; four TTRs (4.7%) were repositioned intraoperatively, and one (1.2%) was subsequently removed and replaced with crushed allograft. All breaches were medial and/or cephalad. Overall, 86 S2AI screws were implanted, ranging from 9.5–10.5 mm in diameter and 80–110 mm in length. No S2AI screws (0%) required repositioning.

**Table 2:** TTRs and S2AI Implant Sizes (diameter × length) for the Combined Cohort

TTR size (mm × mm)	Left	Right	Total
*7.0* × *65*	1	0	1
*7.0* × *70*	2	4*	6
*7.0* × *75*	1	1	2
*7.0* × *80*	12	10^†^	21
*7.0* × *85*	4	3	7
*7.0* × *90*	23	24	47
Total	43	43	86

S2AI size (mm × mm)	Left	Right	Total
*9.5* × *80*	3	1	4
*9.5* × *90*	9	10	19
*9.5* × *100*	25	26	51
*9.5* × *110*	3	3	6
*10.5* × *100*	3	3	6
Total	43	43	86

^*^In 2 cases a 7.0 × 90 mm TTR was initially placed but subsequently replaced with a 7.0 × 70 mm TTR

^†^In 1 case a 7.0 × 80 mm TTR was initially placed but subsequently removed and replaced with allograft

In the initial 21 cases, 3/42 (7.1%) of the TTRs were subject to intraoperative repositioning; this decreased in the subsequent 22 cases to 2/44 (4.5%). Time since the first Bedrock case at our institution was negatively correlated with intraoperative malpositioning, though non-significant (*r* = −0.135; *p* = 0.39). Risk assessment of requiring repositioning in the initial vs subsequent cohort was non-significant (RR = 1.57; *p* = 0.664).

### Malposition cases

**Fig. 5:**
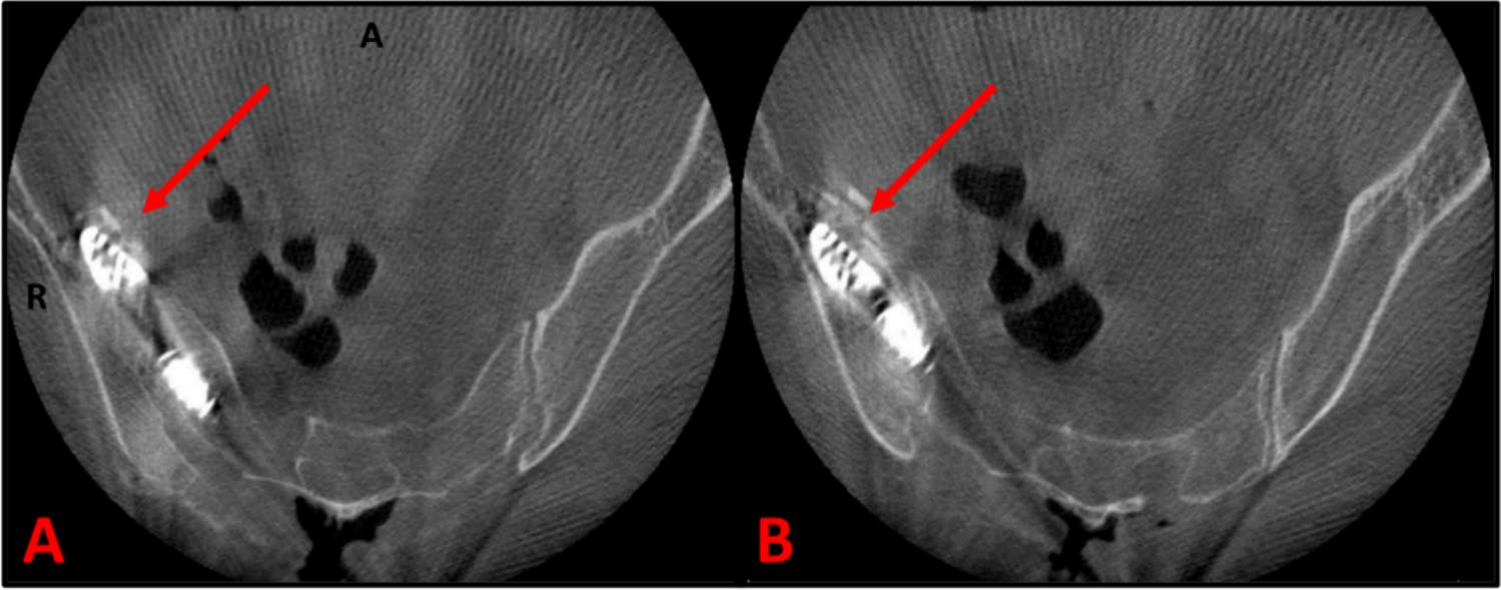
Intraoperative CT scans from malposition case #1 demonstrating: **a** cephalad breach of the right iliac wing by a TTR, **b** fully intraosseous TTR after repositioning laterally

Three patients requiring intraoperative TTR repositioning were described in our initial series [9]. In the subsequent cohort, the first patient (Case #1) was a 59-year-old female with a height of 1.63m and a BMI of 24.75 kg/m^2^. A right medial breach was noted after broaching. The tract was redirected, and a 7.0 × 80 mm TTR was visualized by an intraoperative CT scan to now breach cephalad to the ilium (Fig. 5a). A final tract adjacent to the caudal S2AI screw at the lowest point of the teardrop was successfully made, and the implant was determined to be fully intraosseous (Fig. 5b). Impaction graphing was performed to correct a bony defect in the lateral sacral-ala.

**Fig. 6:**
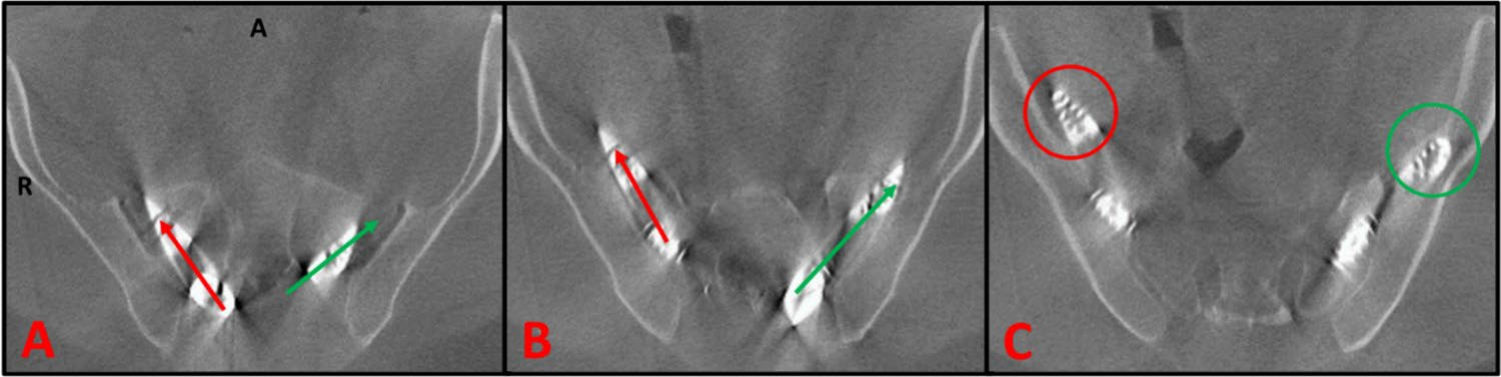
Intraoperative CT scans from malposition case #2 demonstrating a medial breach from TTR skiving (red) and a successful trajectory with iliac purchase (green): **a** Right-TTR skiving within the SI joint after making contact with the hard ilium, **b** right-TTR medial breach into the pelvis, **c** right-TTR distal tip visualized anterior to the iliac wing

The second patient (Case #2) was a 34-year-old male with a height of 1.78m and a BMI of 34.8kg/m^2^. After noting the guide wire tip had broken off during its removal, an intraoperative CT scan showed a medial breach of the right 7.0 × 80 mm TTR without iliac purchase (Fig. 6). Following removal of the implant and broken fragment, a more lateral starting point was used to obtain a good trajectory. However, again upon removal, the tip of the guide wire broke off. Both an intraoperative CT scan and X-ray showed medial TTR migration with the broken guide wire protruding anterior to the ilium. Additional TTR re-insertion was deemed suboptimal, and the implant tract spanning the SI joint was packed with crushed cancellous allograft to induce fusion. The broken guide pin was successfully retrieved anteriorly.

No patients experienced acute complications or required subsequent operations due to malposition. Moreover, patients with intraoperative malposition had no notable neurological deficits, vascular injury, or abdominal visceral injuries.

### Patient reported outcome measures (PROMs)

Mean PROMs for the combined cohort improved significantly at a one-year minimum follow-up for ODI (53.0 vs. 38.1; *p* < 0.001), VAS back pain (5.8 vs. 3.7; *p* = 0.002), and PROMIS-10 physical health scores (10.3 vs. 12.2; *p* < 0.001) (Table 3). PROMIS-10 mental health also improved but did not reach significance (11.4 vs. 12.2; *p* = 0.189). MCID requirements for the combined cohort was met for ODI and VAS back pain. Separately, each cohort (initial/subsequent) resembled the same trends for significance and MCID as the combined cohort; however, VAS back for the subsequent cohort was not significant and did not meet MCID (4.9 vs. 3.0; *p* = 0.056). Between cohorts and between patients with/without malpositioned TTRs, no statically significant differences were found for changes in PROMs (Table 4).

**Table 3:** Combined, Initial, and Subsequent Patient Cohorts’ Comparison of Pre- vs Post-Operative (1-year) PROMs

		Pre-operation (mean ± SD)	1-year post-operation (mean ± SD)	*p* value
Combined Cohort	**ODI** (n = 31)	53.0 ± 13.6	38.1 ± 21.0	< 0.001**
	**VAS Back** (n = 24)	5.8 ± 2.6	3.7 ± 2.7	0.002**
	**PROMIS Mental Health** (n = 29)	11.4 ± 4.1	12.2 ± 4.3	**.189**
	**PROMIS Physical Health** (n = 29)	10.3 ± 2.6	12.2 ± 3.2	< 0.001**
Initial Cohort	**ODI** (n = 17)	56.4 ± 12.7	42.6 ± 18.0	0.002**
	**VAS Back** (n = 12)	6.8 ± 2.1	4.5 ± 2.6	0.014*
	**PROMIS Mental Health** (n = 15)	10.3 ± 2.9	11.5 ± 4.2	.271
	**PROMIS Physical Health** (n = 15)	9.8 ± 2.1	11.3 ± 2.7	0.038*
Subsequent Cohort	**ODI** (n = 14)	48.9 ± 13.9	32.8 ± 23.7	0.009**
	**VAS Back** (n = 12)	4.9 ± 2.7	3.0 ± 2.7	0.056
	**PROMIS Mental Health** (n = 14)	12.5 ± 5.0	12.9 ± 4.4	0.506
	**PROMIS Physical Health** (n = 14)	10.9 ± 3.1	13.2 ± 3.4	0.003**

^**^p value < 0.01**;** *p value < 0.05

**Table 4:** Change in Pre-/Post-Operative (1-year) PROMs Comparison of Initial vs Subsequent Patient Cohorts

	Combined cohort pre/post change (mean ± SD)	Initial cohort pre/post change (mean ± SD)	Subsequent cohort pre/post change (mean ± SD)		*p* value (initial/Subseq.)
ODI	−14.9 ± 17.0†	−13.9 ± 15.0	−16.1 ± 19.7†	0.096	
VAS back	−2.1 ± 2.9†	−2.3 ± 2.7†	−1.9 ± 3.2	0.691	
PROMIS mental health	0.8 ± 3.3	1.2 ± 4.1	0.4 ± 2.3	0.319	
PROMIS physical health	1.9 ± 2.5	1.5 ± 2.6	2.3 ± 2.4	0.575	

^**^p value < 0.01; *p value < 0.05. †met MCID

## Discussion

The Bedrock technique offers a novel method for spinopelvic fixation involving two points of fixation – the first is a traditional sacral-2-alar-iliac (S2AI) screw and the second is a titanium triangular rod (TTR) placed in an SAI trajectory parallel and cephalad to the S2AI screw. Specifically, the TTR was designed with a 3D-printed porous surface and hollow interior to promote osseointegration, thereby leading to fusion of the sacroiliac (SI) joint and creation of a more rigid construct [1, 3, 10–12]. Despite the potentially positive biomechanical implications, literature relating to surgical application is scarce and warranted further investigation. Hence, we aimed to offer insight from our increased experience and to investigate a potential learning curve by comparing our initial and subsequent cohorts.

In our combined cohort of 43 patients, we demonstrated that lumbosacral fusion with concomitant open SI joint fusion using TTRs is technically feasible. Overall, we observed 5.8% (5/86) TTR and 0% S2AI screw malposition rates with four of the five malpositioned cases (80%) being successfully repositioned intraoperatively and one (20%) replaced with allograft. Initial results of a multi-center randomized clinical trial, which compared the TTR Bedrock technique to single S2AI fixation, recently published a 1.8% (4/109) TTR malposition rate [4]. Although lower than our overall malposition rate, we are on track to have a similar rate.

Comparing our two cohorts, we observed a decline in malposition rate from 7.1% in our initial cohort of 21 patients to 4.5% in our subsequent cohort of 22 patients. We found an increased relative risk of malposition in our initial cohort (RR = 1.57), and a negative correlation between malposition rate and time from our institution’s first surgery utilizing the Bedrock technique (r = -0.135). However, neither finding was statistically significant despite having no reported cases of malposition in the last 7 months of the combined cohort. Although greater statistical power is needed, there appears to be a slight decline in the prevalence of malposition using the Bedrock technique at our institution, potentially indicative of a learning curve.

Multiple factors make the placement of a TTRs cephalad and parallel to an S2AI screw challenging. The first challenge presents from differences in the cross-sectional area of the teardrop corridor. As shown in the fluoroscopic teardrop view (Fig. 3), the cross-sectional area is far greater in the most caudal portion but decreases proximally. A retrospective review reported a 42.3% S2AI breach rate for screws placed above the S2 foramina but 0% when placed at or below [14]. Likewise, a recent retrospective study on stacked S2AI screws reported a 4.5% (4/88) cephalad screw malposition rate compared to 0.8% (1/123) for caudal screws [15]. Thus, we suggest positioning the S2AI screw in the caudal most aspect of the teardrop and the TTR just cephalad with the flat edge of the triangular implant facing toward the cylindrical screw to minimize separation (Fig. 3). This is especially important for patients’ with anatomically smaller corridors. Secondly, when crossing the SI joint obliquely, skiving upon contact with the hard iliac bone is a common concern [16]. The blunt shape of the TTR tip compared to the conical tip of screws uniquely predisposes it to skiving. This progression is shown in Fig. 6 for case #2 where the right-sided implant skives medially along the joint while the left side made adequate purchase with the ilium. This is supported by our data which shows all TTR breaches (100%) to be medial and/or cephalad in trajectory. Similarly, initial results of the prospective SILIVA study showed three of its four malpositioned cases to be medial and/or cephalad while the other case was not specified [4]. In an effort to decrease implant skiving, novel fusion devices incorporating a threaded design have been marketed including the iFuse TORQ Implant System designed for SI joint fusion and the iFuse Granite Implant System (SI Bone, Santa Clara, California, USA) with tulips for attachment to posterior rods.

While no studies, to the authors’ knowledge, have directly investigated changes in malposition rates relative to the time of surgeon experience with TTRs or pelvic screws in an SAI trajectory, published results on overall malposition rates for lateral minimally-invasive TTRs and S2AI screws offer a useful comparison. Specifically, comparing our 5.8% TTR malposition rate to our current 0% and previously published 2.6% malposition rate for S2AI screws supports our prediction of a notably higher malposition rate of TTRs compared to screws in an SAI trajectory [15]. However, in comparison to other existing literature on S2AI screw malposition rates, our Bedrock TTRs appeared similar. For example, three institutions, reported ten medial/lateral breaches (7.8%) [17], two medial breaches (1.7%) [18], and one medial breach (5.6%), respectively, all of which were done under CT-guidance [19]. Although these studies corroborate our rate of medial breaching, a single site study using a free-hand technique without guidance reported an 8% malposition rate of which all five moderate-to-severe breaches were lateral [8]. Compared to free-hand, two studies using robotic assistance reported a 4% and 6.3% malposition rate for S2AI screws, although a third study reported no significant difference between techniques [20–22]. Despite variation in technique, malposition rates for S2AI screws were consistently similar to our rate with Bedrock TTRs.

It is important to note that the accuracy of implant placement and respective learning curves may differ across institutions due to surgeon experience and the technology at their disposal. Surgeons at our institution have extensive experience with spinopelvic fixation and refined techniques using S2AI screws. We also use CT-based computer navigation, which has been shown to allow surgeons to more accurately place and assess final placement of spinal implants compared to fluoroscopy [23, 24]. Contrary to what was expected, the initial data from the prospective SILVIA study indicated that 3 of their 4 intraoperative Bedrock TTR breaches were done using navigation; however, only 45% of the 109 cases were done with CT-guided navigation [4]. Given our experience that intraoperative CT scans allow easier detection of implant breaching (Fig. 5), it is possible that these results are contributed to a higher detection rate when using CT-based imaging compared to fluoroscopy. For example, in our initial series, one of the three malpositioned cases was not detected until the time of CT [9].

Although Bedrock TTRs follow a posterior SAI trajectory, TTRs are often used in a lateral minimally invasive approach, which pass across the SI joint more perpendicularly into low density sacral bone [25]. Therefore, we would expect the lateral approach to have a lower skive potential. This is supported by a meta-analysis which reported a 1.4% lateral TTR malposition rate [26]. However, a retrospective study on 90 patients reported a 17.8% malposition rate for percutaneous pin placement, which was noted to affect final TTR position [27]. From our experience, even with robust tracking-frame placement, posteriorly placed TTRs are especially prone to deviation from the guide pin tract as highlighted in Case #2 (Fig. 6). Furthermore, three single-centered studies on lateral SI joint fusions had 5.9%, 5.6%, and 5.0% postoperative TTR malposition rates, respectively [28–30]. Likewise, a multi-center prospective study reported a 6.5% rate using CT scans at 12 months post-operation [31]. However, all five of these studies only identified malpositions post-operatively, which, from our experience, tends to be a lower percentage than those identified intraoperatively. These results are overall comparable to our 5.8% rate of TTR malpositions, despite the greater technical demands of the Bedrock technique.

Concerning learning curves over time, an industry-run study of 11,416 minimally invasive TTR cases noted a decrease in malposition rates over a 5-year period [32]. Likewise, a single surgeon study showed a reduced risk for malposition of CT-guided SI joint fusion rods by 60% over 5 years [33]. Furthermore, ample literature exists showing considerable learning curves for sacral and spinal pedicle screw placement. Although a joint space is not crossed, the small corridor for pedicle screws offers a relative comparison [34]. A three-year retrospective review reported a learning curve for tricortical S1 screw placement using CT-based navigation with 39% successful screw positioning in the first year, 57% in the second, and 91% in the third [17]. Another study utilizing minimally invasive pedicle screws showed a malposition rate drop of 16–8% between the first and fourth year [35]. These studies show steeper learning curves than what we observed with the Bedrock technique (92.9–95.5% accuracy), which was unexpected as this trajectory should theoretically be less prone to skiving. However, it is worth noting that surgeons at our institution have been using navigation for lateral SI joint fusion and S2AI spinopelvic fixation for many years. Also of note, each of these studies were over a much longer time frame than our 2-year series, suggesting a more apparent learning curve for the Bedrock technique if assessed over longer time points.

The efficacy of the Bedrock technique was measured via PROMs, which overall met MCID requirements for mean changes in ODI and VAS back pain scores. Preoperative and 1-year post-operative results showed a favorable change for each PROM with statistically significant improvement for ODI, VAS back, and PROMIS-10 physical health scores (Table 3). There were no notable differences in PROMs between the initial and subsequent cohorts. Long-term outcomes with a greater sample size will be addressed with an ongoing multi-centered prospective study (SILVIA, NCT04062630; clinicaltrials.gov) [4].

### Limitations

We acknowledge that this study is subject to limitations given its relatively small sample size and inclusion of patients from an academic tertiary referral center, which limits the generalizability of our findings. Additionally, patients were treated by a combination of five spine surgeons of differing backgrounds. However, all surgeries were performed in the same location, with the same CT-guided navigation system, and each surgeon has extensive experience with spinopelvic fixation. PROMs were limited to patients with both pre-operative and one-year post-operative reports.

## Conclusion

The objective of this follow-up retrospective review was to report on our increased experience using the Bedrock technique with TTRs, evaluate the potential of a learning curve, and provide strategies for minimizing implant malpositioning. Compared to caudal S2AI screws, with a 0% overall malposition rate, cephalad TTRs had a 5.8% overall malposition rate, of which all were medial/cephalad. Posterior placement of a TTR above an S2AI screw poses technical challenges due to the natural cephalad narrowing of the bony corridor in the teardrop and blunt tip of the implant, predisposing it to medial skiving as it passes through the SI joint. However, this technical challenge was shown to decline slightly between our initial (7.1%) and subsequent (4.5%) cohorts, indicating a potential learning curve. Most influential in reducing malposition was the transition to placing implants as low as possible in the teardrop, thereby increasing the cross-sectional area of the corridor and maximizing available space. In conclusion, the Bedrock technique is technically feasible, and given our descriptive assessment, we hope to help mitigate any associated learning curves.

## Data Availability

No datasets were generated or analysed during the current study.
